# Mass Spectrometry-Based Omics for the Characterization of Triple-Negative Breast Cancer Bio-Signature

**DOI:** 10.3390/jpm10040277

**Published:** 2020-12-12

**Authors:** Ioana-Ecaterina Pralea, Radu-Cristian Moldovan, Adrian-Bogdan Țigu, Corina Ionescu, Cristina-Adela Iuga

**Affiliations:** 1 Department of Proteomics and Metabolomics, Research Center for Advanced Medicine–MedFuture, “Iuliu Hațieganu” University of Medicine and Pharmacy, Louis Pasteur Street 4-6, 400349 Cluj-Napoca, Romania; pralea.ioana@umfcluj.ro (I.-E.P.); moldovan.radu@umfcluj.ro (R.-C.M.); 2 Department of Translational Medicine, Research Center for Advanced Medicine–MedFuture, “Iuliu Hațieganu” University of Medicine and Pharmacy Cluj-Napoca, Louis Pasteur Street 6, 400349 Cluj-Napoca, Romania; bogdan.tigu@umfcluj.ro; 3 Department of Pharmaceutical Biochemistry and Clinical Laboratory, Faculty of Pharmacy, “Iuliu Hațieganu” University of Medicine and Pharmacy Cluj-Napoca, Louis Pasteur Street 6, 400349 Cluj-Napoca, Romania; corina.ionescu@umfcluj.ro; 4 Department of Pharmaceutical Analysis, Faculty of Pharmacy, “Iuliu Hațieganu” University of Medicine and Pharmacy, Louis Pasteur Street 6, 400349 Cluj-Napoca, Romania

**Keywords:** TNBC, mass spectrometry, omics, metabolomics, proteomics, lipidomics

## Abstract

Triple-negative breast cancer (TNBC) represents an unmet medical need due to a high rate of metastatic occurrence and poor overall survival, pathology aggressiveness, heterogeneous clinical behavior and limited cytotoxic chemotherapy options available because of the absence of targetable receptors. The current standard of care in TNBC is represented by chemotherapy and surgery associated with low overall survival and high relapse rates. Hopes of overcoming current limited and unspecific approaches of TNBC therapy lie in studying the metabolic rewiring of these types of breast cancer, thus understanding the mechanisms involved in the occurrence and progression of the disease. Due to its heterogeneity, a clinically relevant sub-classification of this type of breast cancer based on biomarker panels is greatly needed in order to guide treatment decisions. Mass spectrometry-based omics may provide very useful tools to address the current needs of targetable biomarker discovery and validation. The present review aims to provide a comprehensive view of the current clinical diagnosis and therapy of TNBC highlighting the need for a new approach. Therefore, this paper offers a detailed mass spectrometry-based snapshot of TNBC metabolic adjustment, emphasizing a complex network of variables governing the diverse and aggressive clinical behavior of TNBC.

## 1. Introduction

“Triple-negative” refers to a heterogenous and highly aggressive group of breast cancers (BC) that are immunohistochemically characterized by both the lack of estrogen (ER) and progesterone (PR) receptors and the absence of human epidermal growth factor receptor 2 gene (HER2) amplification. Clinically, this term translates into limited and unspecific treatment options (chemotherapy and surgery remaining the standard of care), high relapse rates with metastatic complications (with unknown/undetermined patterns involved in the spread; higher likelihood of brain and lung involvement), and overall low survival rates (hazard ratio (HR) = 1.69, 95%, confidence interval (CI) 1.24–2.30) [1]. 

In the last 30 years, extensive research was conducted to decipher the complex biological behavior and high variability of this type of breast cancer and permit a systematic and effective therapeutic approach. As they have become available, genomic and transcriptomic tools have been applied in an attempt to sub-categorize triple-negative breast cancer (TNBC). Undoubtedly, these techniques expanded our knowledge of TNBC, emphasizing a more complex network of variables and interactions between those that govern the diverse and aggressive clinical behavior already seen in this type of cancer. To date, there is no complete and precise classification of TNBC into distinct clinical and molecular subtypes that could guide treatment decisions, revealing the need for a new perspective.

TNBC should not remain defined by the absence of targetable biomarkers as the current guidelines implies, but should be clinically regarded as a heterogenous group of breast cancers. Moreover, clinical observations and omics studies have emphasized the further need of TNBC sub-classification that could guide a systematic and effective therapeutic approach. 

Mass spectrometry (MS)-based omics have undergone important development in recent years with groundbreaking applications in cancer research, being routinely used in proteomics and metabolomics to investigate a large variety of chemical and biological molecules. 

In the context of personalized medicine, the present study contributes to existing knowledge by offering a comprehensive and critical assessment of TNBC metabolic reprogramming as seen through mass spectrometry-driven omics. Furthermore, this is the first review reporting several MS-based omics approaches (metabolomics, proteomics, lipidomics) offering complementary information of TNBC metabolic adaptations involved in the onset, growth, and recurrence of this type of cancer. 

In order to provide a detailed snapshot of TNBC, a literature review of Embase and PubMed indexed articles was conducted. Emtree and Mesh vocabularies corresponding to “TNBC”, “omics”, “mass spectrometry” key-terms were used when interrogating the databases. Additional studies were identified by searching bibliographies of the selected papers. A subsequent filtration was performed based on the relevance of the data reported in the studies. In the last five years, several reviews on TNBC were published characterizing this type of breast cancer from several points of view: treatment advances and targeted therapies [2,3], and classification strategies based on several biomarkers [4,5]. In contrast, this literature review offers a comprehensive characterization of TNBC, starting with assessing the clinical status and treatment options while emphasizing the current unmet needs of sub-classification, continuing by offering a more detailed TNBC image as it can be observed through MS-based omics, and finishing by providing some perspectives on the clinical diagnosis of TNBC using the aforementioned techniques. 

## 2. Current Clinical Approach of Triple-Negative Breast Cancer (TNBC) 

### 2.1. Breast Cancer Classification Models

Originally, the classification of breast cancers included the tumor size, histological grade and standard immunohistochemistry tests (IHC) status of ER and PR hormone receptors completed later on with the amplification status of HER2. This approach allowed BC to be classified into 3 main groups: hormone sensitive, HER2 positive and TNBC, respectively. Admittedly, the classification had low clinic accuracy: the predicted low-risk patients developed aggressive forms of cancer, while high-risk associated patients had positive responses. The addition of complementary DNA (cDNA) microarray technology allowed for an extended BC classification, able to define four groups with different prognosis and molecular targets, namely: luminal-A, luminal-B, HER2-enriched and basal-like. The underlying concept was that there are two cell types in the mammary gland—luminal and basal cells—that can be differentiated by IHC. Luminal cells express ER and PR and are positive for keratins 8/18, while basal cells are positive for keratins 5/6 and 17 [6]. Although not comprehensive, this classification is still used today by the European Society for Medical Oncology (ESMO) in their clinical practice guidelines for breast cancer diagnosis, treatment and follow-up [7,8] with some additions. In particular, the level of Ki67, a cellular proteic proliferation marker highly expressed in dividing cells (the expression of which in TNBC can reach up to 80%, while in normal mammary tissue it is <3% [6]) with prognostic value only in ER+, HER2-tumors [9]. Additionally, the prognostic value of gene groups (genomic signatures) have been validated through global expression analysis with microarrays and are now commercially available [6]. They provide complementary information to clinicians with the aim of understanding the possible response of a patient to treatment and are highly recommended by ESMO, being classified into two generations based on reverse transcription polymerase chain reaction (RT-PCR) technology used [10]. These gene signatures have limited clinical value, being used only with prognostic value in ER+, HER2- tumors. The first-generation gene signatures were developed on extensive studies upon epithelial cancer cells and their prognostic value can be applied for determining the risk of relapse in ER+ BC, while other types of BC are assigned to the high-risk category [10,11]. In the case of the second-generation gene signatures, changes that occur both in the epithelial cancer cells and myoepithelial and stroma cells were considered. This translated into a better ability to predict the prognosis and late relapse in the case of basal-like and HER2+ molecular subtypes.

### 2.2. Subclassification of TNBC

While there is evidence that TNBC does not overlap with basal-type molecular cancers, the current classification still includes TNBC in the basal-like group [12]. Unquestionably, there is a critical need of molecular definition of TNBC heterogeneity in order to (i) understand the mechanism underlying the diversity of this type of BC, (ii) find specific pathways involved in the observed clinical outcome, (iii) identify molecular signatures that can predict clinical response to therapy with high accuracy, and (iv) develop appropriate targeted therapies for TNBC patients based on the specific shown subtype.

Towards meeting the needs outlined before, several efforts have been made to explore the TNBC heterogeneity. Lehmann et al. published [13] the first sub-classification of TNBC based on gene expression profile. The cluster expression analysis applied revealed six TNBC subtypes displaying distinctive features in terms of RNA expression, somatic mutations, copy-number variations and genes implicated in specific pathways namely: basal-like (BL1 and BL2), immunomodulatory (IM), mesenchymal (M), mesenchymal stem-like (MSL) and luminal androgen receptor (LAR) subtypes. Top gene ontologies for BL1 revealed enhancement of cell cycle–cell division pathways, elevated DNA damage response (ATR/BRCA pathway), and increased Ki67 expression. BL2 subtype presented enriched pathways implicated in growth factor signaling and in glycolysis/gluconeogenesis, while the IM subtype was enriched in immune cell processes. Mesenchymal-like subtypes M and MSL had expression profiles related to cell motility and differentiation. By contrast with the M subtype, MSL presented unique enrichment of genes associated with angiogenesis and stem cells, while low claudin expression and proliferation genes. The LAR subtype was particularly interesting due to enriched expression of genes involved in hormonally regulated pathways, such as steroid synthesis, porphyrin metabolism and estrogen/androgen metabolism, while presenting androgen receptor (AR) expression up to nine times more elevated than the other groups [6]. This subtype also presents the highest expressions of genes involved in amino acid metabolism. In the follow-up study published in 2016, Lehman et al. [14] perfected the TNBC classification algorithm by confirming that the presence of stromal cells in tumors influenced the definition of the IM and MSL subtypes. The authors concluded that the IM status should be determined independently of the subtype due to the potential of the immune-reactive TNBC patients to benefit from immune inhibitors [14]. A strong negative correlation between IM and M subtypes was observed, implying that the mesenchymal TNBC subtype may create an immune suppressive microenvironment. Removal of IM and MSL led to a revised classification including four transcriptional TNBC subtypes: BL1, BL2, M, and LAR (the TNBC type 4 classification) that differ in both clinical and histological characteristics, progression patterns prognosis, chemotherapy response, metastatic recurrences tropism [12,14]. Additionally, the study confirmed that lobular carcinoma is exclusive to the LAR TNBC subtype, while the metaplastic carcinoma can be expected in either M or BL2. Surprisingly, the BL1 tumors presenting a higher grade, are of a lower stage than LAR and associated with an increased patient relapse-free survival. Also, an increased regional lymph node involvement was observed particularly in the LAR subtype associated with bone metastasis, in contrast to the M subtype, where metastasis tropism is targeted to the lungs. Furthermore, about 20% of tested TNBC was classified as immunomodulatory, presenting increased expression of immune checkpoint regulators (PD1, PD-L1 and CTLA4) [14]. This opens the way to a promising therapy direction with PD-1/PD-L1 inhibitors like nivolumab, pembrolizumab, durvalumab, cemiplimab. In relation to the response to neoadjuvant chemotherapy of TNBC subtypes, it was confirmed that BL1 had the highest patient clinical response, while BL2 and LAR had the lowest. These observations emphasize the/a further need for sub-classification of TNBC in order to efficiently identify chemotherapy-responsive and non-responsive patient populations and further optimize therapeutic programs. Recently, Jézéquel et. al. [15] proposed a 3-level classification of TNBC based on the gene expression profiling study conducted in an internal TNBC cohort: one molecular apocrine C1–luminal type with phosphatidylinositol-4,5-bisphosphate 3-kinase catalytic subunit alpha (PIK3CA)-mutated and HER2-enriched phenotype and two basal-like-enriched, biological aggressive C2-with pro-tumorigenic immune response (immune suppressive), characterized by high neurogenesis and C3–anti-tumorigenic, with adaptive immune response and associated with complete B cell differentiation and immune checkpoint upregulation. 

Other classifications have also been proposed [16,17,18], current literature providing profuse evidence to support the need for a personalized approach of TNBC. A first step in this direction could be attained through a comprehensive sub-classification of TNBC in order to provide a powerful and efficient therapeutic treatment based on the targeting of specific metabolic weaknesses. There are several examples of practice observations that sustain the use of certain agents in specific clinical settings e.g.: the gene expression profile confirmed the LAR subgroup of TNBC; this particular subgroup could benefit from anti-androgen therapy (bilucamide, enzalutamide) [8]; PDL-1 overexpressed TNBC could benefit pembrolizumab therapy [19], while for the transmembrane glycoprotein NMB (gpNMB)-overexpressed BC glembatumumab vedotin may represent another promising approach [20]. Moreover, anti-neurogenic therapy could represent a treatment option in C2 TNBC [15].

Nevertheless, the decoding of TNBC molecular subtypes has yet to find applicability in the clinical setting. Overall, to address the current issue of diagnosis and the limited treatment options, TNBC should be regarded as an heterogenous group of breast cancers and not remain defined by absence of targetable biomarkers. Ideally, subgrouping of TNBC should be completed with diagnosis; nonetheless, data regarding specific metabolic alterations, targetable pathways and chemoresistance confirmed by clinical evidence is still pending. 

### 2.3. Diagnosis, Staging and Current Treatment of TNBC

**Figure 1:**
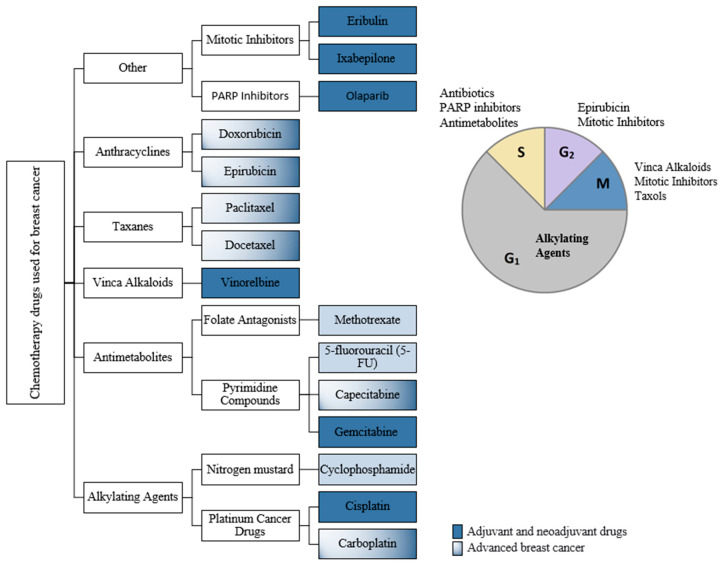
Classification of chemotherapy drugs used in breast cancer (BC) according to the cell-cycle-phase they are active in.

TNBC is highly aggressive and has poor prognosis due to a high tendency to metastasize, high recurrence rates and unstudied heterogeneity. On the other hand, it seems to respond to chemotherapy better than other types of BC [21], Figure 1 presenting a classification of chemotherapy drugs approved for breast cancer. Specifically, for early and non-BRCA-mutated advanced TNBC, chemotherapy alone is the recommended treatment [7,22] although non-specific and highly toxic.

ESMO guidelines [7,8] specify the following: (i) early TNBC should receive chemotherapy; (ii) it is recommended that the primary (neoadjuvant) systemic therapy of TNBC or BRCA1/2-mutated disease to consist of sequential anthracyclines and taxanes and/or platinum; (iii) for advanced TNBC with rapid progression and need of disease control the preferred chemotherapy regimens consist of carboplatin and gemcitabine (or cisplatin) and 5-FU (or capecitabine); (iv) if sequential single agent chemotherapy was selected, the recommended agents belong to the class of anthracyclines or taxanes; (v) depending on the BRCA status, there are other chemotherapy recommendations like: eribulin, vinorelbine, carboplatin. A meta-analysis [23] published on TNBC chemotherapy treatment revealed several trends that may be beneficial in the therapeutic approach:

(i) Neoadjuvant chemotherapy versus adjuvant chemotherapy for TNBC patients: 

Neoadjuvant chemotherapy has provided certain benefits in the management of systemic micro-metastases, tumor burden and detection of tumor sensitivity to chemotherapeutic drugs [23]. The meta-analysis conducted by Xia et al. [23] aimed to examine existing evidence regarding survival of TNBC patients with neo-adjuvant (before surgery instituted chemotherapy) versus adjuvant therapy regimens. Several observations were reported: i. low overall survival rate was associated with neo-adjuvant chemotherapy (HR = 1.59; 95% CI = 1.25–2.02; *P* = 0.0001); ii. no statistically significant difference in disease-free survival of TNBC patients between the two treatments (HR = 0.85; 95% CI = 0.54–1.34; *P* = 0.49); iii. patients reaching pathological complete response with neoadjuvant chemotherapy had radically improved overall (HR = 0.53; 95% CI = 0.29–0.98; *P* = 0.04) and disease-free survival rates (HR = 0.52; 95% CI = 0.29–0.94; *p* = 0.03); iv. in contrast, patients with residual disease after neoadjuvant chemotherapy had worse overall and disease-free survival rates than those receiving adjuvant chemotherapy (HR = 1.18; 95% CI = 1.09–1.28; *P* < 0.0001 and HR = 2.36; 95% CI = 1.42–3.89; *P* = 0.0008, respectively) [23].

(ii) Addition of platinum-based agents to the chemotherapy regimen of TNBC patients:

Poggio et al. [24] were interested in the efficacy and safety impact of platinum-based neoadjuvant chemotherapy in TNBC. In the systematic review published in 2018, they included nine randomized controlled trials and determined the odds ratio (OR) and hazard ratio (HR) (95% CI) for pathological complete response, event-free survival, overall survival and risky adverse effects of platinum-based neoadjuvant chemotherapy. Statistics confirmed that this strategy significantly increased pathological complete response (OR 1.96, 95% CI 1.46–2.62, *p* < 0.001) [24] while significant risk of hematological side effects were associated with the platinum agent addition to the standard anthracycline- and taxane-based therapies. 

Further investigation of platinum chemotherapy in neoadjuvant and metastatic settings confirmed pathologic complete response after neoadjuvant treatment for early TNBC (OR = 1.75; 95% CI = 1.36–2.26) while not statistically significant progression-free survival rate in metastatic TNBC [25]. Moreover, addition of platinum-based drugs to taxanes regimen showed substantial increase in progression pathological complete response rate in comparison with platinum + anthracycline regimen (44.6% vs. 27.8%) [25]. 

The BRCA gene is critical in DNA repair and loss of function may result in an increased sensitivity to alkylating agents such as platinum cancer drugs in the BRCA-mutated TNBC. This theory was tested by Caramelo et al. [26] in their recently published meta-analysis comprising seven studies (a total of 808 TNBC patients, 159 were BRCA mutated). The trend showed that when compared to wild-type TNBC patients, addition of platinum to chemotherapy regimens increases pathologic complete response rate in BRCA-mutated TNBC (OR = 1.459 CI 95% = [0.953–2.34] *p* = 0.082) [26]. Admittedly, due to the small number of patients included, no statistical significance was achieved. Thereupon, the need to positively confirm the utility of platinum compounds in this setting is still pending.

(iii) Addition of targeted agents in the management of TNBC:

The beneficial influence of targeted agents alone or in addition to chemotherapy in both early and metastatic TNBC settings was assessed in multiple meta-analysis published in the last five years. Due to these analyses, drug classes as antimetabolites (capecitabine), poly(ADP-ribose) polymerase (PARP) inhibitors (olaparib, talazoparib), monoclonal anti-bodies for inhibition of vascular endothelial growth factor (VEGF) or PD-L1 (known as immune checkpoint inhibitors) are now being tested in clinical trials for TNBC. A recent review paper published by Nakhjavani et al. [27] develops this topic and presents current development of targeted therapy for TNBC. The authors offer a comprehensive report of molecular targets in TNBC emphasizing new, promising therapies being presently in clinical testing. In the subsequent section, cases of such targeted agents are being presented.

Capecitabine represents the prodrug of 5-fluorouracil. It has been used as second-line chemotherapy in gastric and metastatic breast cancer when patients had previously received an anthracycline and taxane regimen [28]. The impact of adding capecitabine in an early TNBC setting to anthracycline and taxane chemotherapy upon survival rate was the focal point of Li et al. meta-analysis published in 2020 [28]. The subsequent conclusions yielded: Disease-free survival rate with addition of capecitabine to standard chemotherapy had an HR  =  0.77, 95% CI = 0.66–0.90 while for overall survival rate supporting capecitabine supplement the study reported an HR  =  0.69, 95% CI = 0.56–0.85. Improvement was substantial, particularly when 6 to 8 cycles of the drug were administered. In terms of adverse effects, hand-foot syndrome and diarrhea were reported in capecitabine groups (OR  =  51.08, 95% CI = 9.02–289.22, *p*  <  0.001 and OR  =  5.00, 95% CI = 2.00–12.53, *p* <  0.001 respectively). Instead, lower rates of vomiting (OR  =  0.52, 95% CI = 0.32–0.85, *p* =  0.008) were associated with the administration of capecitabine [28].

The effectiveness and safety of bevacizumab addition to chemotherapy in managing metastatic breast cancer setting was assessed by Li et al. in their publication [29]. Bevacizumab is a recombinant humanized monoclonal antibody that binds to VEGF, important inducer of vasculogenesis. The drug has been granted market permissions in the European Union (EU) since 2005 being used in combination for the treatment of metastatic and recurrent forms of colon, non-small cell lung cancer, kidney, cervix, ovary and fallopian tube and breast cancers [30]. In the TNBC metastatic cases (1312 patients), reported progression-free survival was considerably enhanced by the addition of bevacizumab to standard chemotherapy with an HR of 0.61, 95% CI: 0.47–0.80, *P*  <  0.001 [29]. Atezolizumab, a humanized immunoglobulin G1 monoclonal antibody that directly binds to PD-L1 with reactivation of the antitumor immune response [31], is the first checkpoint inhibitor approved in TNBC by both FDA and EMA organisms [3,32].

Network meta-analysis data [20,33,34] also revealed improvement of pathologic complete response and progression-free survival rates when chemotherapy regimens containing taxanes were supplemented by bevacizumab. Pembrolizumab-containing regimens were also correlated with a high pathologic complete response rate [33]. The beneficial effects of the addition of other molecules to the chemotherapy scheme were emphasized. Chen et al. [20] showed by a Bayesian meta-analysis that iniparib and bevacizumab supplementation of chemotherapy can increase the overall survival of TNBC patients. Moreover, in terms of progression-free survival rate, the addition of cetuximab, orafenib, bevacizumab, veliparib, iniparib, ipatasertib and olaparib to chemotherapy can be advantageous [20]. Conversely, when reporting to the metastatic setting of TNBC, only bevacizumab and veliparib were discovered beneficial [34]. Supplementation of chemotherapy with ipatasertib, cetuximab, iniparib, and sorafenib may well improve the overall survival rate of TNBC patients regardless the stage of the cancer [20].

## 3. Mass Spectrometry-Based Omics in TNBC 

### 3.1. Metabolomics

Metabolic reprogramming is regarded as a hallmark of cancer that sustains the abnormal development of malignant cells. Therefore, understanding the metabolic adaptations and new dependences of cancer cells may provide key tools in the therapeutic approach. TNBC represents an unmet medical need with no defined milestones to overcome problems to overcome, thus being relatively stagnant in terms of therapeutic approach for years as previously described [2,3]. Advances in the development of large-scale “omics” have mass spectrometry as core technology. Understanding biological systems needs an integrated study at multiple levels of control: transcription and mRNA degradation, protein dynamics and post translational modifications and metabolite concentrations and fluxes. Modern mass spectrometry-based omics provide efficient means of addressing current needs in handling this disease: sub-classification, metabolic vulnerabilities, new, specific and efficient therapeutic targets, early detection means. 

The most comprehensive MS-based metabolomics study on TNBC cell lines was published by Beatty et al. [35]. The group studied the metabolome of the breast cancer cell lines in an attempt to subcategorize the TNBC and discover potential targets that can be therapeutically exploited. Twelve TNBC cell lines representative of the six Lehman et al. [14] TNBC subtypes were included in the MS-based metabolome profiling study along with the MCF10A breast epithelial cell line and two independent isolates of primary human mammary epithelial cells (HMEC), as controls [12,14]. The unsupervised hierarchical clustering analysis, conducted on 155 metabolites, clustered the cell lines into 3 metabolic subtypes (MST1-control cell lines, MST2 and MST3-TNBC cell lines). Fatty acid metabolites including docosahexaenoic acid, arachidonic acid, and gamma-linolenic acid were reported as increased in TNBC vs. control cell lines, while branched chain amino acids (valine and leucine) as well as one aromatic amino acid (tryptophan) had decreased levels. In TNBC, particularly in the MST2 metabolic subtype, lower glutathione and its precursors was reported, demonstrating that TNBC cell lines exhibit metabolome adjustments consistent with elevated oxidative stress. Furthermore, the same study highlighted different sensitivity to glutathione biosynthesis inhibition by buthionine sulphoximine (BSO) across the triple negative cancer cell lines panel: the MST2 subtype showed high sensitivity to BSO, while MST3 and the control subtype showed intermediate sensitivity and resistance.

Yang et al. [36] used untargeted and stable isotope-assisted gas chromatography mass spectrometry (GC-MS) metabolomics to characterize MDA-MB-231 changes in molecular mechanisms under hypoxic conditions. It is well known that hypoxia is a factor that promotes further cancer development due to activation of hypoxia-inducible factor 1-alpha (HIF-1α) and changes induced in the primary energy sources of the cancer cells [36,37]. The study of Yang et al. [36] reported low levels of glucose, pentose phosphate pathway and tricarboxylic acid (TCA) intermediates and of the amino acids apart from serine associated with MDA-MB-231 cell line under hypoxia. Also, fatty acid metabolism was significantly downregulated, while high levels of glucose-6-phosphate, serine, lactate, 2-hydroxyglutarate and metabolites for nucleotide synthesis were reported under the hypoxic condition. The hypoxia TNBC cell line presented lower glucose levels and increased levels of glycolysis-related metabolites (glucose-6-phosphate, glucose-1-phosphate, lactate), emphasizing an upregulated glycolysis. Under hypoxia, the TNBC cell line favored glutamine to glucose as its TCA cycle main source, showing low levels of a-ketoglutarate, fumarate and malate. Both glucogenic and aromatic amino acids were downregulated in the hypoxic condition, while glutamine-derivate glutamate and serine was found increased in these conditions. Concerning the lipid metabolism, cholesterol and fatty acids metabolism were decreased in TNBC cell lines as a response to hypoxic conditions [36]. 

There is evidence of TNBC clinical non-conformity among different races and ethnic groups [38]. Moreover, large invasive tumors with high node positivity and histologic grade are more commonly seen in Asian patients. Starting from these observations, Li et al. [39] performed a liquid chromatography mass spectrometry (LC-MS)-based metabolomic study on serum samples from 31 TNBC Asian patients. 77 metabolites significantly altered compared to control. The glycerophospholipids were significantly dysregulated, while cardiolipins were downregulated in TNBC samples. Additionally, TNBC samples had upregulated amino acid levels, namely, leucine, proline, threonine, tyrosine, valine, pyroglutamic acid and N-acetyl-L-histidine [39]. N-acetyl-L-histidine and octanoylcarnitine were uniquely reported as significant metabolites associated with TNBC in the Asian population. The pathway enrichment analysis emphasized glycerophospholipid metabolism, aminoacyl-tRNA biosynthesis, and valine, leucine, and isoleucine biosynthesis as the most significant altered metabolic pathways. Admittedly, several pathways mostly implicated in lipid metabolism (glycerophospholipid metabolism, alpha-linolenic acid, fatty acid metabolism), amino acid metabolism (glycine, serine, threonine, phenylalanine, tyrosine and tryptophan) and the TCA cycle were significantly altered in the serum of the TNBC patients [39].

### 3.2. Lipidomics

Lipids are essential molecules involved in surface chemistry and interfacial catalysis, the production of signaling molecule precursors or signaling by acting themselves. Moreover, lipids are actively implicated in the cell energy housekeeping and regulate pathways involved in energy homeostasis e.g. regulation of the activity of nuclear transcription factors: peroxisome proliferator-activated receptors (PPARs), nuclear factor-κB (NF-κB) and sterol regulatory element-binding proteins (SREBPs) [40]. Glycerophospholipids (GPL) act as signaling molecules and have shown involvement in the regulation of migration, apoptosis and neurotransmission, while diacylglycerols (DG) are regarded as second messengers involved in apoptosis and mediation of signal transduction in cancer cells [41]. Nevertheless, altered expression of enzymes involved in lipid metabolism (synthesis, storage, activation and degradation) has been reported in breast tumors [42].

Several studies have shown alterations in the expression of enzymes involved in lipid metabolism [42,43,44]. Additionally, metabolomics study of the microenvironment of tumor cells have shown that after interaction with breast cancer cells, cancer-associated adipocytes reprogram their metabolism involving upregulation of almost all macronutrients—carbohydrates, lipids, and amino acids [45,46]. Conversely, limited research on lipidomics of breast cancer is available and even fewer studies have addressed TNBC, although altered lipid metabolism represents a well-established hallmark of cancer development. Eghlimi et al. [47] employed LC-MS/MS for the assessment of 110 lipids in plasma of TNBC and control patients. Although conducted on a small number of samples (166 plasma samples: 45 controls, 96 non-TNBC, and 25 TNBC), the study allowed the construction of 2 biomarker panels capable of distinguishing TNBC and early-stage TNBC from controls and TNBC (early stage TNBC) from non-TNBC, respectively. The area under the receiving operating characteristic curve (AUROC) associated with each panel was AUROC = 0.93, sensitivity = 0.96, specificity = 0.76 for TNBC vs Controls and AUROC = 0.96, sensitivity = 0.95, and specificity = 0.89 for ES-TNBC vs Controls. Furthermore, a pathway enrichment analysis was performed on the significant lipids (*p* < 0.05) distinguishing TNBC from controls. The enrichment analysis highlighted choline metabolism, sphingolipid signaling and glycerophospholipid metabolism as being dysfunctional in this type of cancer. There are several conclusions of the study that should be noted: (i) a significant role of sphingolipids in maintaining TNBC progression was highlighted by the presence of elevated ceramide levels in TNBC plasma: Cer 36:1(2), Cer 42:2(1), Cer 43:1, Cer 44:2, Cer 42:3, and Cer 38:1(2); (ii) certain phosphatidylcholines (PC) had inconsistent levels: upregulation of PC 32:1 and PC 34:1 and a downregulation of PC 40:2 was observed; (iii) other PC class representatives were essential in TNBC differentiation from non-TNBC namely, ↑PC 40:3, ↑PC 39:8; ↓PC 34:0; ↓PC 38:9 and TNBC from control: ↓PC 40:2, ↑PC 32:1; (iv) several diarylglycerolipids (DG) were also found to be essential in differentiation between TNBC and control: DG 34:2; DG 36:4(1), DG 32:0, DG 34:1, DG 36:1, DG 36:2(5), DG 38:4 with a certain trend of downregulation in TNBC [47]. 

A comprehensive lipidomics study was performed by Eriksson et al. [41] on several breast cancer cell lines including 2 representatives of TNBC (MDA-MB-231 and MDA-MB-436 with BRCA1 mutation) and MCF10A as control. The study concluded that there is an increased phosphatidylcholine synthesis correlated with TNBC cell lines: DG 32:0 and DG 34:0 were significantly more abundant in TNBC cell lines, while a decreased level of TG was also observed in these cell lines. Also, increase levels of PC ≥ C-40 in TNBC cell lines were reported [41]. 

### 3.3. Proteomic Signature of TNBC

**Figure 2:**
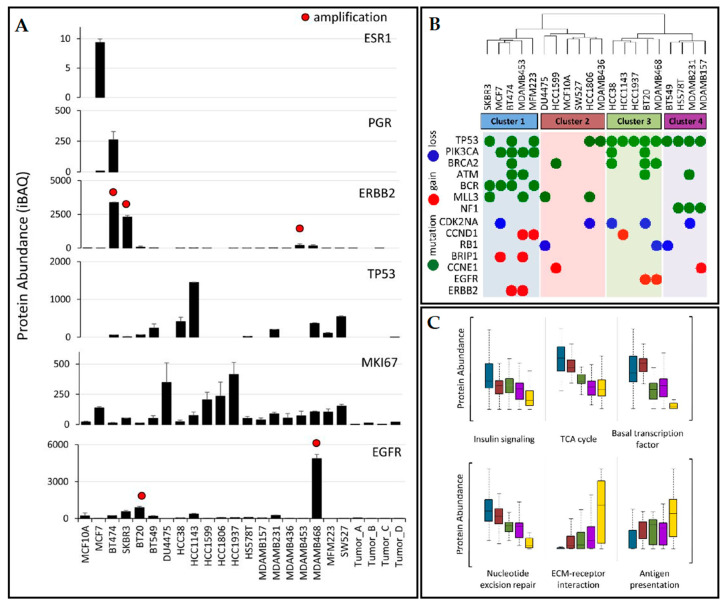
(**A**) Clinical biomarkers quantification in the tested cell lines; (**B**) hierarchical clustering of protein expression profiles with genetic irregularities superimposed; (**C**) protein abundances dissemination within clusters for indicated biological processes; cluster membership is indicated by the same colors used in (B), with tumor samples indicated in yellow. ESR1, estrogen receptor; PGR, progesterone receptor; ERBB2, human epidermal growth factor receptor-2; TP53, tumor protein p53; MKI67, Ki-67 antigen; EGFR, human epidermal growth factor receptor. Absolute protein abundance was calculated using iBAQ. Error bars represent standard deviation. Red dots indicate > 7 gene copy-number amplification. (Partially reproduced with permission from ref. [48]).

The most thorough proteomics study on TNBC cell lines was performed by Lawrence et al. in 2015 [48]. The group performed a quantitative proteomics analysis of 20 human-derived breast cell lines (16 triple-negative cell lines encompassing mesenchymal, luminal, and basal-like subtypes from the Lehmann et al. [13] classification, as well as three receptor-positive and one non-tumorigenic cell line) and four primary breast tumors, having, as the main objective, an in-depth characterization of the proteogenomic landscape of TNBC [48]. The cell lines were examined by several clinical breast cancer biomarkers as seen in Figure 2. 

Hierarchical clustering was applied to the proteomics profiles of the cell lines (Figure 2B) and these were classified into 4 subtypes; data were superimposed with gene alterations. Cluster 1 contained luminal breast cancer type cell lines including the luminal-androgen-receptor cell line. The second cluster was similar to basal-like 2 and also contained the normal breast epithelial cell line MCF10A, while the third cluster had basal-like 1 cell lines (HCC38, HCC1143, HCC1937, BT20, and MDA-MB-468). Finally, the fourth cluster (BT549, HS578T, MDA-MB-231, and MDA-MB-157) was identical to “mesenchymal-like/claudin-low” Lehmann et al. subtype. Several observations were made: (i) PIK3CA and BCR mutations association with luminal breast cancer subtypes (cluster 1); (ii) TP53 mutations specific to TNBC (all cell lines in clusters 3 and 4 presented this mutation); (iii)mutations in the tumor suppressor NF1 were exclusive to the mesenchymal-like subtype (cluster 4). Proteins characterized by significant variation in level of expression within each cluster subtype were reported: STAT5A (uniprot id P42229) was highly expressed in mesenchymal cell lines, FOXA1 (uniprot id P55317) was reported as exclusively expressed in luminal-like cells, while PPM1A (uniprot id P35813) involved in TGF-beta signaling regulation was decreased in TNBC. Instead, proteins involved in immunity and metastasis were significantly upregulated in TNBC cells: POSTN (Uniprot id Q15063), MYLK (Uniprot id Q15746), HLAA (Uniprot id P04439). Furthermore, pathways associated with metastasis, such as ECM-receptor interaction, cell adhesion, and angiogenesis were reported as upregulated in TNBC. Other observations comprise: ephrin type A receptors are overexpressed in many TNBC cell lines, although they are involved in embryonic development and not normally present in adult tissues; isoform-specific protein expressions were revealed for the transcription factor NF-κB, the tumor antigen CD47, and focal adhesion kinase PTK2. The dataset provided by this study is a useful resource to further exploit the biology on TNBC. 

Metabolic flexibility displayed by TNBC assures selective advantage for survival in hostile conditions: it has been recently shown the capacity of glycolysis to OXPHOS switching, surrounding cells metabolic cancer induced changes, endocytosis amplification. Several studies have now focused on identifying and characterizing the function of proteins which are overexpressed in several malignancies, while absent or present at low levels in normal tissues. Blomme et al. [49] investigated myoferlin expression across 51 breast cancer cell lines of different molecular subtype. They reported an overexpressed level of myoferlin in TNBC cell lines and attributed to the protein essential function in regulation of the fatty acid household being important for the growth of TNBC xenografts in vivo. Identifying proteins associated with recurrence was the main objective of Pedersen et al. group [50]. Based on this observation, Ternette et al. [51] studied potential targets for immunotherapy development by immunopeptidome profiling of patient tumors and adjacent normal tissues. Sample preparation ensured accurate HLA-associated peptide purification from the biopsy material. Cofilin-1, IL-32, PCNA, syntenin-1, and ribophorin-2 were reported as a potential panel for therapeutic targets [51]. Faktor et al. [52] were interested in the membrane proteins associated with migration and metastasis. Accordingly, the authors applied high-resolution quantitative mass spectrometry with stable isotope labeling (SILAC) to analyze the surfaceome on the MDA-MB-231 cell line. Desmocollin-1 (highly expressed at the surface of migration-competent MDA-MB-231 sub-population) and catechol-o-methyltransferase were reported as proteins associated with breast cancer cell migration and metastasis. MS-based proteomics has provided valuable tools in TNBC cell line subtyping, protein profile expression in TNBC vs. control studies, identification of metabolic alteration specific to different TNBC development-stage (early onset, metastatic, recurrent) and provided possible protein therapy-targets. 

Despite the success of the large-scale “-omic” studies in providing further understanding of molecular drivers of TNBC (Table 1 presents a succinct MS-based omics description of TNBC metabolic adaptations), several limitations have been emphasized: protein expression patterns are highly cell-line specific highlighting the validity of cell line representativeness of the tumor cellular component and the importance of further selection in cancer research; there are clearly differences driven by the stage of TNBC and intrinsic invasiveness despite overall concordance of whole proteome profiles with various cellular phenotypes. Therefore, large cohort studies of TNBC and corresponding normal tissue/serum are needed to address subtyping and staging of TNBC, the generation of drug sensitivity prediction resource and biomarker discovery.

## 4. Perspectives of Implementation of MS-Based Methods for TNBC Diagnosis

Mass spectrometry-based diagnosis has seen an unprecedented development in the last decade, with certified IVD instruments being offered by some of the biggest manufacturers in the field and, at the same time, an increasing number of certified diagnostic kits are commercially available for a series of diseases. During the same period, regulatory legislation has evolved around the world (e.g., European Union (EU) Regulation 746/2017), paving the way to personalized medicine approaches. 

Mass spectrometry has a well-established role in clinical proteomics, as described in a recent review by Macklin et al. [54]. In cancer research, proteome profiling is the most promising approach, but for now high-resolution mass spectrometry still lacks in many aspects, such as high costs, the required expertise (to run the instruments and analyze the data) and standardization [54,55]. To overcome some of these aspects, some revolutionary MS-based instruments are under development, such as the iKnife [56] and the MasSpec Pen [57], both used for intra-operatory diagnosis. The implementation of these kinds of device could also have a tremendous impact in the diagnosis of breast cancer and, therefore, a better classification and characterization of each type of cancerous tissue is mandatory. TNBC could benefit of all these emerging technologies, but only after defining a correct fact-based classification and a complete proteomic, lipidomic and metabolomic characterization of the tissue types. In such a case, the technology for lipidomic and metabolomic MS-based profiling is already mature enough for clinical use and, considering the pace of development of proteomics tools, soon proteomic diagnosis of TNBC could be available. Until then, efforts have to be put into understanding all the different TNBC subtypes.

## 5. Conclusions

Proteomics, metabolomics and lipidomics studies have provided essential information regarding: (i) the means for sub-classification of TNBC through pathway modifications (glycolysis augmentation, fatty acid and amino acids metabolism adaptation, mitochondrial oxidative metabolism shifting, enrichment of immune-modulatory pathways) responsible for the malignant transformation, drug resistance, and stage-specific cell phenotype (early-onset, malignant, invasive); (ii) potential protein biomarkers for targeted therapeutic approaches (Fatty acid synthase (FASN), Phosphoglycerate dehydrogenase (PHGDH), Glucose Transporter (GLUT), glutaminase (GLS), Cofilin-1); (iii) evidence of isoform-specific protein expression of TNBC as a significant regulatory mechanism in cancer; (iv) metabolic interaction of TNBC and the microenvironment, especially with adipocytes. Admittedly, these studies have also drawn attention to the need for proper selection of cancer models for in vitro omics studies and for larger cohort data (comparative control vs. TNBC) to address the unmet clinical need: the sub-classification of TNBC. The presented MS-based omics tools could identify specific patterns to address the heterogeneity of TNBC. Moreover, these tools may find applications in targeted and more personalized therapy of this aggressive form of breast cancer. 

## Figures and Tables

**Table 1:** Mass spectrometry (MS)-based omics description of triple-negative breast cancer (TNBC) metabolic adaptations.

	Sample Type	Affected Pathways (TNBC vs. Control)	Ref.
Metabolomics and lipidomics	Patient plasma	Lipid metabolism Choline metabolism Sphingolipid signaling Glycerophospholipid metabolism	[47]
	Patient serum	Lipid metabolism Glycerophospholipid metabolism Alpha-linonelic acid Fatty acid metabolism Amino acid metabolism Valine, leucine, and isoleucine biosynthesis	[39]
	Cell line(hypoxia)	Amino acid metabolism Glycine, serine and threonine metabolism Alanine, aspartate, and glutamate metabolism Aminoacyl-tRNA biosynthesis phenylalanine metabolism Glutathione metabolism D-glutamine and D-glutamate metabolism Pyruvate metabolism Pentose phosphate pathway	[36]
	Cell line (MUC-1 glycoprotein expression)	Amino acid metabolism Metabolism of arginine and proline, alanine, aspartate, and glutamate D-glutamine and D-glutamate metabolism	[53]
	Cell lines	Lipid metabolism Glutathione metabolism Amino acid metabolism	[35]
Proteomics	Cell lines	Amino acid metabolism Signal transduction pathways TGF-β-signaling pathways Vehicle mediated transport Gap junction trafficking and regulation Cell adhesion signaling pathway Focal adhesion Development biology Axon guidance pathway	[48]
	Cell lines	Endocytosis of fatty acids	[49]
	TFFE breast cancer tissues (Recurrence)	MHC class I antigen-presentation Cell cycle pathway	[50]
	Breast tissue	MHC class I antigen-presentation	[51]
	Cell line (migration capabilities)	Metabolism of amine-derived hormones Mediation of cell-cell adhesion	[52]
